# Treatment outcomes of retinal vein occlusion in clinical practice in Nepal

**DOI:** 10.1186/s12886-021-01857-y

**Published:** 2021-02-18

**Authors:** Sanjeeb Bhandari, Manish Poudel, Indira Paudyal, Pratibha L. Joshi, Chunu Shrestha, Govinda Paudyal, Eli Pradhan

**Affiliations:** 1 The University of Edinburgh, Edinburgh, UK; 2 Tilganga Institute of Ophthalmology, Kathmandu, Nepal; 3 Eye see you Eye care centre, Kathmandu, Nepal

**Keywords:** Retinal vein occlusion, Treatment outcomes, Bevacizumab

## Abstract

**Background:**

This study evaluated the treatment outcomes of retinal vein occlusion (RVO) in a routine clinical practice in Nepal.

**Methods:**

This was a retrospective analysis of observational data of patients with RVO who attended the retina clinic of the Tilganga Institute of Ophthalmology from 1 November 2017 to 31 October 2018. The main outcome was the mean change in visual acuity (VA) at 12 months from the start of treatment. Other outcomes of interest were the mean change in central subfield thickness (CST) and the number of treatments over 12 months.

**Results:**

A total of 99 eyes (of 99 patients) with RVO (60 - branch RVO [BRVO] and 39 - central RVO [CRVO] were available for the analysis. Eyes with CRVO had worse VA and CST at baseline. Eyes in both groups were similar for age, associated factors for RVO, duration of vision loss and the presence of ischemia at baseline. The mean (95% Confidence Interval [CI]) VA change at 12 months for BRVO was − 0.35 (− 0.46, − 0.23) logMAR (*p* < 0.001) from a mean (SD) of 0.75 (0.42) logMAR at baseline with 63% achieving VA < 0.3 logMAR while for CRVO it was − 0.35 (− 0.46, − 0.23) logMAR (*p* = 0.19) from 1.13 (0.61) logMAR at baseline and VA < 0.3 logMAR in 36%. The mean (95% CI) change in CST over 12 months was − 114 (− 189, − 40) μm (*p* = 0.003) from a mean (SD) of 423 (151) μm at baseline for BRVO and − 184(− 276, − 91) μm (*p* < 0.001) from 519 (213) μm for CRVO. Patients in both groups received a median of 2 bevacizumab injections over 12 months. Around 37% eyes were lost before 12 months’ observation. The mean VA and CST trajectory in these eyes at their last visit was similar to those that completed 12 months.

**Conclusion:**

The outcomes of RVO over the 12 months were inferior and the number of treatments fewer than those of the clinical trials and other reports from routine clinical practice. Future studies to identify the treatment barriers are warranted to improve the treatment outcomes in our patients.

## Background

The prevalence of retinal vein occlusion (RVO), a common cause of vision impairment often associated with cardiovascular or other systemic diseases, in Nepal is on the rise [1–3]. The cause for the impaired vision in eyes with RVO (branch retinal vein occlusion [BRVO] and central retinal vein occlusion [CRVO]) is macular edema and ischemia [4]. Treatment options for RVO includes laser photocoagulation and intravitreal injections of either vascular endothelial growth factor (VEGF) inhibitors or steroids. The Branch Retinal Vein Occlusion Group during the 1980’s reported that laser photocoagulation improved vision in eyes with BRVO [5]. The pivotal phase 3 randomised clinical trials (RCT) of intravitreal injections of VEGF inhibitors for RVO, from the earlier half of this decade, reported good visual and anatomic outcomes than those reported with macular laser photocoagulation and intravitreal injections of triamcinolone [6–11].

The VEGF inhibitors are the preferred first line agents in the treatment of RVO [12, 13]. The choice of VEGF inhibitors among countries varies according to their drug regulatory processes, reimbursement policies and its availability. Bevacizumab, owing to its cost, is the preferred VEGF inhibitor for the treatment of macular edema due to BRVO and CRVO in Nepal. Studies reporting the treatment outcomes of RVO in Nepal are limited. Such studies in routine clinical practice provides data which can be used to improve the outcomes where they appear suboptimal and in formulating treatment guidelines. This study evaluated the treatment outcomes in eyes with RVO in routine clinical practice in Nepal.

## Methods

This was a retrospective analysis of data collected from patients ≥18 years of age with RVO who attended the retina clinic of the Tilganga Institute of Ophthalmology from 1 November 2017 to 31 October 2018 thereby allowing the possibility of having at least 12 months of observations after the initial treatment. Treatment naïve eyes of patients who met the above selection criteria and started bevacizumab for the treatment of macular oedema secondary to RVO were included in the analysis. Eyes of patients with macular oedema secondary to causes other than RVO (such as diabetic macular oedema, central serous chorioretinopathy, uveitis) and those with concomitant corneal diseases that could cause vision impairment (such as corneal opacities) were excluded. Data recorded at each visit included duration of decreased vision, best corrected visual acuity (VA) read on the Snellen chart (converted to logarithm of minimum angle of resolution [logMAR]), intraocular pressure (Goldman applanation tonometry), central subfield thickness (CST [μm]) measured using spectral domain optical coherence tomography (OCT), neovascularization of the retina, iris or angle, ocular procedures, gonioscopy and adverse events. Duration and type of diabetes, hypertension, dyslipidemia, presence of cardiovascular diseases was recorded at the first visit (baseline). Fluorescein angiogram was done at the discretion of the treating physician as were macular laser for BRVO and sectoral/panretinal photocoagulation for neovascularization or its prophylaxis. All treatment decisions, including choice of treatment and frequency of visits, were based on VA and OCT at the discretion of the practitioner in consultation with the patient thereby reflecting real-world clinical practice. Informed consent was sought from the patients. The Institutional Review Board of the Tilganga Institute of Ophthalmology approved the study. This study adhered to the tenets of the Declaration of Helsinki.

The main outcome was the mean change in VA at 12 months. The mean change in CST and number of treatments were also evaluated. The proportions of eyes with VA ≤ 0.3 logMAR (20/40 Snellen equivalent) at 12 months and those with VA ≥1 logMAR (20/200) were calculated. The proportion that gained ≥ 3 lines of vision and those that lost similar lines at 12 months were also evaluated. Outcomes were also analysed in eyes stratified by baseline VA into two groups, good vision (≤ 0.3 logMAR) and > 0.3 logMAR (20/50), to study the relationship of baseline VA on the VA change. The VA and CST outcomes in eyes that were lost before 12 months were also evaluated.

### Statistictics

Descriptive data included the mean, standard deviation (SD), 95% confidence interval (CI), median (first and third quantiles, Q1 and Q3) and percentages where appropriate. Changes at 12 months were tested using independent t-test, Wilcoxon-Mann-Whitney test, Chi-square test and Fisher's Exact test as appropriate. The last observation was carried forward to report outcomes in eyes that were lost before 12 months. A generalized additive model was used to display VA and CST in eyes with 12 months follow-up. Locally estimated scatterplot smoothing (LOESS) curves, a non-parametric strategy for fitting a smooth curve to data points, were used to display the relationship of VA and CST from the baseline to the last observed visit in eyes that were lost before 12 months. All analyses were performed using R version 4.0.1 (http://www.R-project.org/) with the mgcv package (V1.8–33) for generalized additive (mixed) model computation.

## Results

### Study population

There was a total of 120 eyes (120 patients) with RVO in the period specified. Of these, 21 eyes that also had concomitant diseases for vision impairment were excluded thus, only 99 eyes were available for the analysis. Sixty eyes (60.6%) had BRVO while 39 (39.4%) had CRVO. Participants had a mean (SD) age of 56 (15) years. The mean (SD) duration of vision loss was 70 (86) days. Associated risk factors were documented in most (81 patients, 82%) of them at baseline. The more frequent individual associated factors were hypertension in 59 (60%), smoking in 27 (27%), diabetes in 22 (22%), hyperlipidemia in 19 (19%) and alcohol consumption in 10 (10%). The proportion of patients with these factors were similar in both CRVO and BRVO groups (Table 1). Eyes with CRVO had worse vision (VA: 1.13 logMAR [6/75 Snellen equivalent] versus 0.75 logMAR [6/30] for BRVO, *p* = 0.001) and macular thickness (CST: 519 μm versus 423 μm, *p* = 0.02) at baseline than those with BRVO. Table 1 gives an overview of baseline characteristics of eyes in the two groups.

**Table 1:** Demographic characteristics

	CRVO	BRVO	*p* value
Patients, n	39	60	
Female, n (%)	17 (44)	35 (58)	
Right Eye, n (%)	26 (67)	32 (53)	
Hypertension, %	51	65	0.25
Diabetes, %	18	25	0.56
Hyperlipidaemia, %	15	22	0.6
Smoking, %	28	27	1
Alcohol, %	10	10	1
Glaucoma, %	0.1	0	0.15
Vision loss, days mean (SD)	82 (78)	114 (144)	0.16
Age, years mean (SD)	53 (17)	58 (13)	0.09
Baseline VA logMAR, mean (SD)	1.13 (0.61)	0.75 (0.42)	0.001
VA ≤ 0.3 logMAR, %	10	20	0.31
VA ≥ 1 logMAR, %	59	37	0.04
Baseline IOP mm Hg, mean (SD)	19 (11)	16 (3)	0.07
CST μm, mean (SD)	519 (213)	423 (151)	0.02
Presence of Ischaemia			
Ischaemic, %	18	20	1
NonIschaemic, %	82	80	

*n* Number, *SD* Standard Deviation, *VA* Visual Acuity, *CST* Central Subfield Thickness, *logMAR* logarithm of minimum angle of resolution

### Visual outcomes at 12 months

The mean (SD) VA in the RVO cohort 0.6 (0.61) logMAR (20/80 Snellen equivalent) at 12 months improved from the baseline of 0.89 (0.53) logMAR (20/168 Snellen equivalent, *p* < 0.001). The mean (95% confidence interval) VA change at 12 months was − 0.29 (− 0.4, − 0.18) logMAR. The mean VA in eyes with good initial vision (≤0.3 logMAR, 20/40 Snellen equivalent or better) dropped from the baseline while it improved in those presenting with VA > 0.3 logMAR (20/50 or worse). The proportion of eyes with VA ≤ 0.3 logMAR (20/40 or better) increased to 53% at 12 months from 16% at baseline (*p* = 0.02) while those with VA ≥ 1 logMAR (20/200 or worse) decreased to 24% from 45% at baseline (*p* < 0.01).

**Fig. 1:**
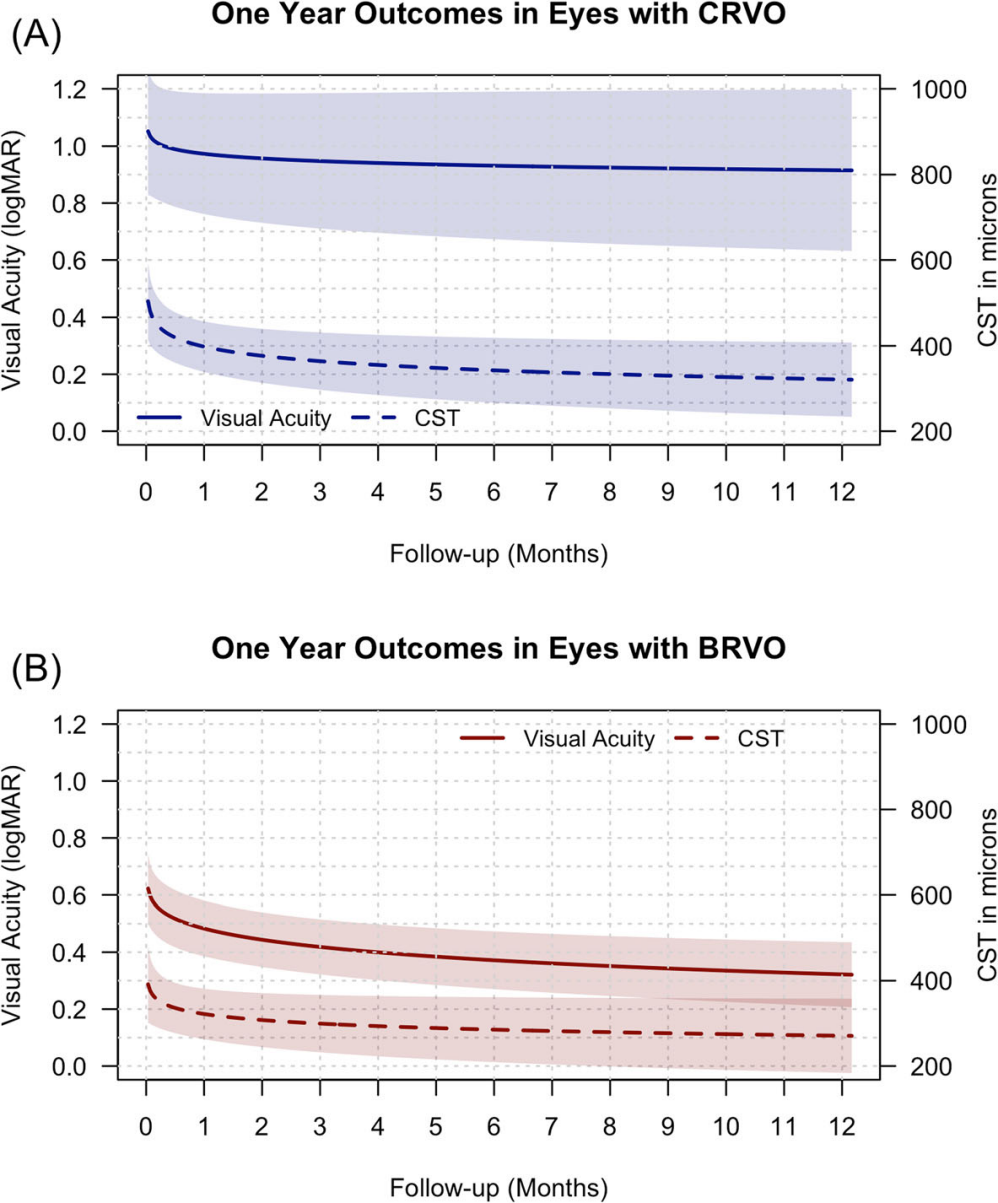
Line-graphs showing the mean visual acuity (solid line) in logMAR (y-axis) and central subfield thickness (CST, dashed line) in microns (z-axis) over 12 months in eyes with central retinal vein occlusion (CRVO, blue) (**a**) and branch retinal vein occlusion (BRVO, brown) (**b**) that completed 12-month observation. The shaded area represents the 95% confidence interval

Figure 1 illustrates the mean VA over 12 months in eyes with CRVO and BRVO that completed 12 months’ observation. The mean (95% CI) VA change at 12 months in BRVO, − 0.35 (− 0.46, − 0.23) logMAR, was significant (*p* < 0.001) but those of CRVO, − 0.2 (− 0.43, 0.02) logMAR, was not (*p* = 0.19). The proportion of eyes with VA ≤ 0.3 logMAR increased and those with VA ≥ 1 logMAR decreased in both groups but more so in eyes with BRVO (Table 2).

**Table 2:** One-year outcomes

	CRVO	BRVO	*p* value
Patients, n	39	60	
Baseline VA logMAR, mean (SD)	1.13 (0.61)	0.75 (0.42)	0.001
Final VA logMAR, mean (SD)*	0.92 (0.75)	0.4 (0.4)	< 0.001
Change VA logMAR*, mean (95% CI)	−0.2 (− 0.43, 0.02)	−0.35 (− 0.46, − 0.23)	0.25
VA ≤ 0.3 logMAR, % (baseline/final*)	10 / 36	20 / 63	0.01
VA ≥ 1 logMAR, % (baseline/final*)	59 /47	37 /12	< 0.001
Improvement ≥3 lines*, %	32	60	0.37
Worsening ≥3 lines*, %	8	7	0.31
Baseline IOP mmHg, mean (SD)	19 (11)	16 (3)	0.07
Final IOP mmHg, mean (SD)*	17 (2)	15 (5)	0.05
Change IOP mmHg*, mean (95% CI)	−3 (−6, 0)	−2 (−4, −1)	0.64
Baseline CST μm, mean (SD)	519 (213)	423 (151)	0.02
Final CST μm, mean (SD)*	343 (160)	281 (133)	0.09
Change CST μm*, mean (95% CI)	−184 (−276, −91)	− 114 (− 189, −40)	0.24
Injections ^α^, median (Q1, Q3)	2 (1, 3)	2 (1, 3)	1
Additional Macular Laser, n	0	2	0.52
Additional Triamcinolone, n	2	2	0.65
Additional PRP/Sectoral Laser, n	16	15	0.17

*n* Number, *VA* Visual Acuity, *SD* Standard Deviation, *CI* Confidence Interval, *CST* Central Subfield Thickness, *Q1* First Quantile, *Q3* Third Quantile, *PRP* Panretinal photocoagulation, * Last observation carried forward for lost to follow-up, ^α^ Number of bevacizumab injections

We divided the cohort into two subgroups according to the VA at baseline, eyes with VA ≤ 0.3 logMAR and those with VA > 0.3 logMAR, to study the relationship of the initial VA on VA gains with treatment. Eyes with good initial vision (VA ≤ 0.3 logMAR) had a minimal mean (95% CI) VA change at 12 months, 0.03 (− 0.13, 0.2) logMAR from a mean (SD) of 0.22 (0.25) logMAR at baseline (*p* = 0.68) in BRVO and 0.26 (− 1.04, 1.57) logMAR from a mean (SD) of 0.28 (0.05) logMAR at baseline (*p* = 0.57) in CRVO. The mean (95% CI) VA change in eyes with initial vision > 0.3 logMAR was greater in BRVO, − 0.44 (− 0.57, − 0.32) logMAR from 0.89 (0.34) logMAR at baseline (*p* < 0.001), but was not so for CRVO, − 0.26 (− 0.49, − 0.03) logMAR from 1.22 (0.57) logMAR at baseline (*p* = 0.1). However, the final vision in the initial VA > 0.3 logMAR subgroup in BRVO, despite the higher gain, was lower than in the good initial vision group (Table 3).

**Table 3:** Outcomes stratified by Visual Acuity at presetation

	CRVO (*n* = 39)	BRVO (*n* = 60)	*p* value
Eyes with presenting VA ≤0.3 logMAR			
Eyes, n (%)	4 (10)	12 (20)	0.05
Baseline VA logMAR, mean (SD)	0.28 (0.05)	0.18 (0.13)	0.5
Final VA logMAR, mean (SD)*	0.54 (0.83)	0.22 (0.25)	0.71
Change VA logMAR*, mean (95% CI)	0.26 (−1.04, 1.57)	0.03 (−0.13, 0.2)	0.61
Baseline CST μm, mean (SD)	423 (152)	330 (102)	0.31
Final CST μm, mean (SD)*	268 (50)	230 (110)	0.46
Change CST μm*, mean (95% CI)	− 155 (− 390, 80)	− 79 (− 206, 48)	0.42
Injections^α^, median (Q1, Q3)	0.5 (0, 1.5)	0 (0, 1.2)	0.94
Additional Macular Laser, n	0	0	1
Additional Triamcinolone, n	0	0	1
Additional PRP, n	1	5	1
Eyes with presenting VA ≤0.3 logMAR			
Eyes, n (%)	35 (90)	48 (80)	
Baseline VA logMAR, mean (SD)	1.22 (0.57)	0.89 (0.34)	0.02
Final VA logMAR, mean (SD)*	0.97 (0.74)	0.44 (0.42)	< 0.001
Change VA logMAR*, mean (95% CI)	−0.26 (−0.49, −0.03)	−0.44 (−0.57, − 0.32)	0.15
Baseline CST μm, mean (SD)	531 (218)	449 (153)	0.07
Final CST μm, mean (SD)*	355 (168)	291 (137)	0.12
Change CST μm*, mean (95% CI)	− 188 (− 294, −82)	−120 (−206, −34)	0.30
Injections^α^, median (Q1, Q3)	2 (1, 3)	2 (1, 3)	0.72
Additional Macular Laser, n	0	2	0.5
Additional Triamcinolone, n	2	2	1
Additional PRP/Sectoral Laser, n	15	10	0.05

*n* Number, *VA* Visual Acuity, *SD* Standard Deviation, *CI* Confidence Interval, *CST* Central Subfield Thickness, *Q1* First Quantile, *Q3* Third Quantile, *PRP* Panretinal photocoagulation, * Last observation carried forward for lost to follow-up, ^α^ Number of bevacizumab injections

### Macular thickness

Bevacizumab significantly reduced the mean CST in eyes with RVO (Fig. 1). The mean (95% CI) change in CST at 12 months was − 145 (− 203, − 88) μm from a mean of 460 (183) μm at baseline (*p* < 0.001). The mean (95% CI) change in CST at 12 months in CRVO, − 184 (− 276, − 91) μm (*p* < 0.001), and in BRVO, − 114 (− 189, − 40) μm (*p* = 0.003), was similar (0.24, Table 2). Eyes in both VA subgroups had significant reduction in the mean CST after 12 months of treatment (Table 3).

### Treatments

The median (Q1, Q3) number of bevacizumab injections in eyes with RVO over 12 months was 2 (1, 3). Less than two-fifths (36%) eyes required additional treatments, including laser (macular and panretinal photocoagulation/sectoral) and intravitreal steroid (triamcinolone) injections, during the 12 months (Table 2). None of the eyes that received an additional steroid continued further treatment with steroid. The median number of bevacizumab injections in CRVO and BRVO were (2 [1, 3]) versus (2 [1, 3], *p* = 1). There was no difference in the median number of bevacizumab injections when eyes were stratified based on the initial VA, however, more eyes with CRVO in the initial vision > 0.3 logMAR group received laser photocoagulation (*p* = 0.05 , Table 3).

### Non-completion rate at 12 months

**Fig. 2:**
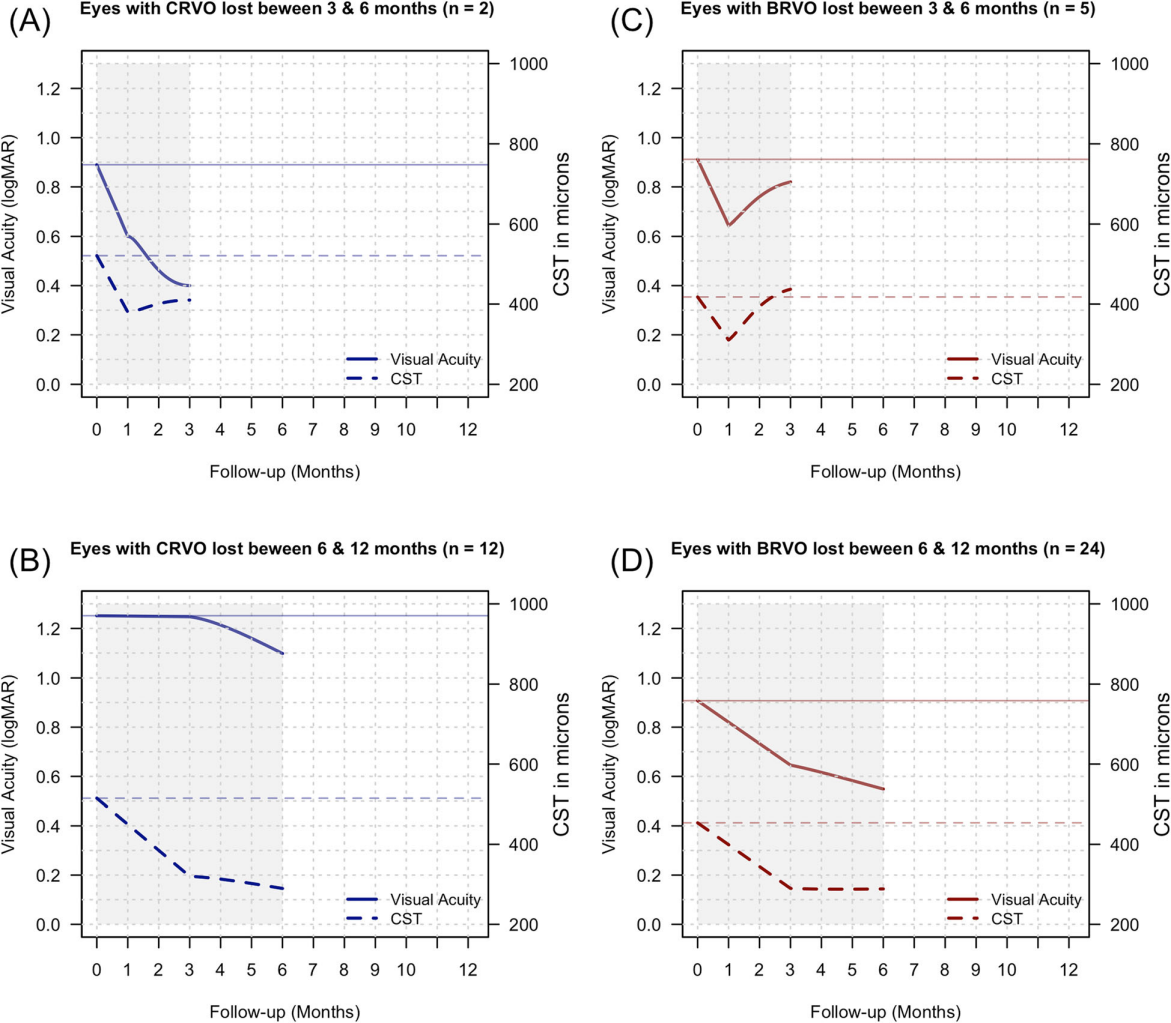
Locally estimated scatterplot smoothing (LOESS) curves showing change in visual acuity and central subfield thickness (CST) over time in eyes of patients that were lost to follow-up grouped by follow-up time; 2 patients with central retinal vein occlusion dropped out between 3 and 6 months (**a**) while 10 did between 6 and 12 months (**b**). Of those with branch retinal vein occlusion, 5 patients dropped out between 3 and 6 months (**c**) and 19 between6 and 12 months (**d**). The shaded region indicates area of progressive decrease in the number of patients, resulting in increased volatility of LOESS predictions

Thirty-seven eyes (37%, CRVO – 12 and BRVO – 25 eyes, *p* = 0.37) were lost before the 12 month’s visit. The mean (95% CI) VA change from the start of treatment to their last visit was − 0.36 (− 0.55, − 0.2) logMAR for BRVO (*p* < 0.001) and − 0.31 (− 0.56, − 0.06) logMAR for CRVO (*p* = 0.01) while the CST change was − 98 (− 222, 26) μm for BRVO (*p* = 0.11) and − 204 (− 384, − 25) μm for CRVO (*p* = 0.03). These eyes received a median of 2 bevacizumab injections, median (Q1, Q3) of 2 (1, 2) for BRVO and 2 (1, 3) for CRVO injections. Figure 2 illustrates the mean VA and CST trajectory of CRVO and BRVO eyes from baseline to their last observed visit before the dropout.

### Adverse events

Of the 21 eyes with non-ischemic CRVO at baseline that completed 12-month observation, 5 eyes (24%) converted to ischaemic CRVO. None of the 24 eyes with non-ischemic BRVO at baseline that completed 12-month visit had ischaemic conversion. Neovascularisation of the retina was observed in 21 eyes (CRVO – 11 [28%], BRVO – 10 [17%], *p* = 0.26). Of these, 2 eyes with CRVO (1 with tractional retinal detachment and vitreous haemorrhage [VH] at 12 months and the other with VH at 6 months) and 1 eye with BRVO (that had VH at 6 months) required a vitrectomy. Twenty-three eyes (59%) with CRVO developed iris neovascularisation over 12 months of which, 7 had angle neovascularisation. Glaucoma was controlled with medications in 9 eyes while 2 eyes required a trabeculectomy. Injection related complications, such as uveitis, cataract progression, retinal detachment and endophthalmitis, was not observed over 12 months.

## Discussion

We found that patients with BRVO in our clinical practice had improved vision and reduced macular thickness at one year while vision improvement in those with CRVO, despite the reduction in macular thickness, was not significant. Patients with RVO that had good initial vision, VA ≤ 0.3 logMAR (20/40), maintained this level of vision while those with VA > 0.3 logMAR (20/50) gained a mean of 0.4 logMAR. Eyes received a median of 2 bevacizumab injections over the 12 months. Thirty-seven eyes (37%) were lost before 12 months. The mean VA and CST trajectory in these eyes from the start of treatment to the last observed visit was similar to those that completed 12- month observation. They received a median of 2 bevacizumab injections before they dropped out. Neovascularization on the retina was simialr for both BRVO and CRVO. Almost three-fifth eyes with CRVO had neovascularization of the iris and a third of these also had neovascularization at the angles. Injection related complications, such as endophthalmitis, were not observed during the study.

The mean reduction in CST at 12 months with bevacizumab in the present study was similar for both BRVO and CRVO. The anatomical outcome mirrored functional outcome in BRVO however, it did not for CRVO which was also observed in a study that evaluated the outcomes of ranibizumab for RVO in routine clinical practice in Denmark [14]. The mean VA which was worse at baseline in CRVO in the present study remained so at 12 months which was similar to those reported in other observational studies of RVO [15–17]. There were fewer eyes with VA ≤ 0.3 logMAR (20/40) and more with VA ≥ 1 logMAR (20/200) at 12 months in CRVO than in BRVO. The degree of macular edema and the subsequent photoreceptor damage could have limited visual recovery in CRVO despite the improvement in the mean CST.

Visual outcomes we observed were strongly related to the VA at presentation. Eyes with initial VA ≤ 0.3 logMAR, had much lower net gain perhaps due to the ceiling effect and fewer treatments, but maintained better vision at 12 months (VA ≤ 0.3 logMAR in 63% for BRVO and 36% for CRVO). Those presenting with VA > 0.3 logMAR had higher gains with improvement towards the mean. Eyes in the good VA group received fewer injections than those in the VA > 0.3 logMAR group.

Studies have found that the visual outcomes of eyes receiving VEGF inhibitors for retinal diseases in routine clinical practice are inferior to those of clinical trials [18–21]. The mean VA of patients at baseline in the present study was worse than those in the pivotal clinical trials of VEGF inhibitors for BRVO, The Ranibizumab for the Treatment of Macular Edema following Branch Retinal Vein Occlusion: Evaluation of Efficacy and Safety (BRAVO) study [6], and Intravitreal Aflibercept for Macular Edema following Branch Retinal Vein Occlusion (VIBRANT) study [7], and CRVO, Ranibizumab for the Treatment of Macular Edema after Central Retinal Vein Occlusion: Evaluation of Efficacy and Safety (CRUISE) study [8], VEGF Trap-Eye: Investigation of Efficacy and Safety in Central Retinal Vein Occlusion (COPERNICUS) study [10] and VEGF Trap-Eye for Macular Edema Secondary to Central Retinal Vein Occlusion (GALILEO) study [9]. Patients with BRVO in the present study that had VA better than 0.3 logMAR (6/12) at 12 months was similar to those of BRAVO and VIBRANT as was those that gained ≥3 lines of vision [6, 7]. Patients with CRVO that gained ≥3 lines of vision in the present study however, were fewer than those in CRUISE, COPERNICUS and GALILEO [8–10]. The mean VA at 12 months we observed however, was inferior to those of the pivotal clinical trials [6–10]. Patients in the present study received 2 bevacizumab injections over 12 months in contrast to the 9 VEGF inhibitor injections in the pivotal clinical trials. Besides the regimen, the choice of the VEGF inhibitor in our cohort might have contributed to the suboptimal outcomes than those reported in the clinical trials.

The drop in macular thickness over 12 months we observed were lower than reported in the clinical trials [6–10]. The maculae of the eyes at baseline in the present study were thinner than those in the clinical trials [6–10]. Patients did not receive bevacizumab injections when eyes had good vision (VA ≤ 0.3 logMAR) irrespective of the thickened maculae as shown by the median number of treatments in the subgroup that had initial VA ≤ 0.3 logMAR. This partly explains why our patient received fewer treatments and suboptimal anatomical outcomes than those of the clinical trials [6–10].

The mean change in VA in BRVO at 12 months we observed was similar to a meta-analysis that reported outcomes of VEGF inhibitor treatments for BRVO in routine clinical practice [21]. The mean drop in CST at 12 months in the present study was inferior and intravitreal treatments fewer than those reported in the meta-analysis [21]. The deterioration of macular anatomy precedes functional impairment when VEGF inhibitor treatments are withheld in RVO [22]. Treatments in the present study were withheld when vision was good, which could have led to the fewer treatments and the thickened maculae in our cohort. The visual and anatomic outcomes of CRVO at 12 months in the present study were inferior and the number of treatments fewer than those reported in studies in routine clinical practice [15–17]. Neovascularization on the retina and iris in our cohort was more frequent than reported in those studies. Neovascularization and the need of panretinal laser may be unnecessary in eyes that receive adequate treatment for vascular retinal diseases [23, 24].

In addition to the failure to treat when the VA was better, other factors could have contributed to the fewer treatments observed in our cohort. The cost of the repetitive bevacizumab treatments as observed for cataract surgery in the population could have affected the treatment adherence [25]. Patients’ perception of visual improvement could have affected their willingness to continue treatment [26]. Poor treatment compliance may be due to other co-morbidities which might have received more attention than the eye disease.

High participants dropout is an unfortunate feature in observational studies. Almost 37% of eyes in the present study dropped out before the 12-month observation. Most of these were lost after 6 months of observations. The mean VA and CST in these eyes were better than the baseline before they dropped out. We do not know whether these eyes dropped out because they felt they were seeing well and did not want to continue injections, or they may have stopped due to a poor outcome.

This study has some limitations that are inherent to observational studies. The treatment decisions in the study, in contrast to clinical trials, were made without adjudication from a reading centre or reference to study protocols. The VA transferred from Snellen chart to logMAR may be less reliable than those obtained using a logMAR chart [27]. The cost of bevacizumab treatment, which are borne by the patient, could have influenced the treatment decision and follow-up visits. Transfer of some patients to their nearest facility could have also led to the high drop outs in the present study. Nevertheless, this study provides data on the treatment outcomes of RVO in our clinical practice.

## Conclusion

Our findings indicate that the availability of VEGF inhibitors may not of itself produce the best outcomes possible. The improvement in VA and anatomic outcomes over 12 months in eyes with RVO in the observational cohort we studied were inferior to those reported in the pivotal clinical trials and observed in routine clinical practice elsewhere. Our outcomes suggest deficiencies in the management and treatment of RVO patients which could be likely affected by lack of implementation of optimal diagnostic and retreatment criteria. Such suboptimal outcomes can result in severe impairment of quality of life of the individual. Future analysis should aim to identify the barriers so that the treatment outcomes of our patients could be improved.

## Data Availability

Data can be shared upon request.

## Abbreviations

RVO: Retinal vein occlusion
BRVO: Branch retinal vein occlusion
CRVO: Central retinal vein occlusion
VEGF: Vascular endothelial growth factor
RCT: Randomised clinical trial
VA: Visual acuity
logMAR: Logarithm, of the minimum angle of resolution
CST: Central subfield thickness
OCT: Optical coherence tomography
SD: Standard deviation
CI: Confidence interval
Q: Quantiles
LOESS: Locally estimated scatterplot smoothing
LOCF: Last observation carried forward
BRAVO: The Ranibizumab for the Treatment of Macular Edema following Branch Retinal Vein Occlusion: Evaluation of Efficacy and Safety study
VIBRANT: Intravitreal Aflibercept for Macular Edema following Branch Retinal Vein Occlusion study
CRUISE: Ranibizumab for the Treatment of Macular Edema after Central Retinal Vein Occlusion: Evaluation of Efficacy and Safety study
COPERNICUS: Vascular Endothelial Growth Factor Trap Eye: Investigation of Efficacy and Safety in Central Retinal Vein Occlusion Study
GALILEO: Vascular Endothelial Growth Factor Trap Eye for Macular Edema Secondary to Central Retinal Vein Occlusion Study
